# Omicron Waves in Argentina: Dynamics of SARS-CoV-2 Lineages BA.1, BA.2 and the Emerging BA.2.12.1 and BA.4/BA.5

**DOI:** 10.3390/v15020312

**Published:** 2023-01-22

**Authors:** Carolina Torres, Mercedes Nabaes Jodar, Dolores Acuña, Romina Micaela Zambrana Montaño, Andrés Carlos Alberto Culasso, Ariel Fernando Amadio, Paula Aulicino, Santiago Ceballos, Marco Cacciabue, Humberto Debat, María José Dus Santos, María Florencia Eberhardt, Carlos Espul, Fabián Fay, María Ailén Fernández, Franco Fernández, Juan Manuel Fernandez Muñoz, Florencia Ferrini, Fernando Gallego, Adriana Angélica Giri, Agustina Cerri, Elisa Bolatti, María Ines Gismondi, Stephanie Goya, Iván Gramundi, José Matías Irazoqui, Guido Alberto König, Viviana Leiva, Horacio Lucero, Nathalie Marquez, Cristina Nardi, Belén Ortiz, Luis Pianciola, Carolina Beatriz Pintos, Andrea Fabiana Puebla, Carolina Victoria Rastellini, Alejandro Ezequiel Rojas, Javier Sfalcin, Ariel Suárez, Estefanía Tittarelli, Rosana Toro, Gabriela Vanina Villanova, María Cecilia Ziehm, María Carla Zimmermann, Sebastián Zunino, Proyecto PAIS Working Group, Laura Valinotto, Mariana Viegas

**Affiliations:** 1 Instituto de Investigaciones en Bacteriología y Virología Molecular (IbaViM), Facultad de Farmacia y Bioquímica, Universidad de Buenos Aires, Ciudad Autónoma de Buenos Aires 1113, Argentina; 2 Consejo Nacional de Investigaciones Científicas y Técnicas (CONICET), Ciudad Autónoma de Buenos Aires 1425, Argentina; 3 Laboratorio de Virología Hospital de Niños Dr. Ricardo Gutiérrez, Ciudad Autónoma de Buenos Aires 1425, Argentina; 4 Instituto de Investigación de la Cadena Láctea (IDICAL) INTA-CONICET, Rafaela 2300, Argentina; 5 Laboratorio de Biología Celular y Retrovirus, Hospital de Pediatría “Prof. Juan P. Garrahan”, Ciudad Autónoma de Buenos Aires 1245, Argentina; 6 Hospital Regional Ushuaia; Universidad Nacional de Tierra del Fuego, Antártida e Islas del Atlántico Sur, UNTDF y CADIC-CONICET, Ushuaia 9410, Argentina; 7 Instituto de Biotecnología/Instituto de Agrobiotecnología y Biología Molecular (INTA-CONICET), Hurlingham 1686, Argentina; 8 Instituto de Patología Vegetal-Centro de Investigaciones Agropecuarias Instituto Nacional de Tecnología Agropecuaria (IPAVE-CIAP-INTA), Córdoba 5020, Argentina; 9 Instituto de Virología e Innovaciones Tecnológicas (INTA-CONICET), Hurlingham 1686, Argentina; 10 Laboratorio de Diagnóstico-UNIDAD COVID- Universidad Nacional de Hurlingham, Hurlingham 1688, Argentina; 11 Laboratorio de Salud Pública, Godoy Cruz 5501, Argentina; 12 CIBIC Laboratorio, Rosario 2000, Argentina; 13 Laboratorio Central Neuquén, Ministerio de Salud de la Provincia del Neuquén, Neuquén 8302, Argentina; 14 Instituto de Medicina y Biología Experimental de Cuyo (IMBECU—CONICET), Mendoza 5500, Argentina; 15 Laboratorio de Medicina Genómica, Facultad de Medicina, Universidad Nacional del Nordeste, Corrientes 3400, Argentina; 16 IBR-CONICET, Facultad de Ciencias Bioquímicas y Farmacéuticas, Universidad Nacional de Rosario, Rosario 2000, Argentina; 17 Departamento de Ciencias Básicas, Universidad Nacional de Luján, Luján 6700, Argentina; 18 Instituto de Medicina Regional, Resistencia 3500, Argentina; 19 Departamento de Biología y Genética Molecular, IACA Laboratorios, Bahía Blanca 8000, Argentina; 20 Laboratorio de salud pública, Facultad de Ciencias Exactas, UNLP, La Plata 1900, Argentina; 21 Laboratorio de Biotecnología Acuática, Facultad de Ciencias Bioquímicas y Farmacéuticas, Universidad Nacional de Rosario, Centro Científico Tecnológico y Educativo Acuario del río Paraná, Rosario 2000, Argentina; 22 Laboratorio de Virología Molecular, Hospital Blas L. Dubarry, Mercedes 6600, Argentina; 23 PAIS Working Group, Ministerio de Ciencia Tecnología e Innovación, Ciudad Autónoma de Buenos Aires 1425, Argentina

**Keywords:** SARS-CoV-2, Omicron, variants, evolution, South America, dynamics, BA.1, BA.2, BA.4, BA.5

## Abstract

The COVID-19 pandemic has lately been driven by Omicron. This work aimed to study the dynamics of SARS-CoV-2 Omicron lineages during the third and fourth waves of COVID-19 in Argentina. Molecular surveillance was performed on 3431 samples from Argentina, between EW44/2021 and EW31/2022. Sequencing, phylogenetic and phylodynamic analyses were performed. A differential dynamic between the Omicron waves was found. The third wave was associated with lineage BA.1, characterized by a high number of cases, very fast displacement of Delta, doubling times of 3.3 days and a low level of lineage diversity and clustering. In contrast, the fourth wave was longer but associated with a lower number of cases, initially caused by BA.2, and later by BA.4/BA.5, with doubling times of about 10 days. Several BA.2 and BA.4/BA.5 sublineages and introductions were detected, although very few clusters with a constrained geographical distribution were observed, suggesting limited transmission chains. The differential dynamic could be due to waning immunity and an increase in population gatherings in the BA.1 wave, and a boosted population (for vaccination or recent prior immunity for BA.1 infection) in the wave caused by BA2/BA.4/BA.5, which may have limited the establishment of the new lineages.

## 1. Introduction

The COVID-19 pandemic has strongly impacted all populations worldwide and was lastly driven by the Omicron variant, causing new waves of infections in almost all regions of the world since the end of the year 2021. Molecular surveillance has been encouraged, especially since the emergence of SARS-CoV-2 variants, and has become an important tool to help prevent the COVID-19 burden when those results were used for public health purposes.

The diversity and evolution of SARS-CoV-2 are reflected by both variants and lineages. While variants of concern (VOCs), namely Alpha, Beta, Gamma, Delta and Omicron, have been defined by the World Health Organization to prioritize the monitoring of some groups of SARS-CoV-2 sequences [1], thousands of lineages have been defined under the Pango system to track the viral transmission and spread more in detail [2].

In particular, Omicron sequences have been classified into lineages BA.1 to BA.5, as well as several sublineages or derived lineages, which may present some biological or clinical differences. For instance, BA.2 cases showed lower or similar risks of death or hospital admission than BA.1 [3,4], and these lineages showed no differences in vaccine effectiveness or in the rate of immunity decline over time [5]. In addition, evasion of neutralizing antibodies was found for BA.2.12.1 and BA.4/BA.5, compared with BA.2, against vaccinated individuals or individuals with immunity elicited by BA.1 [6,7].

In contrast, South America suffered the first Omicron wave almost simultaneously with other regions in December 2021–March 2022, and since then, other Omicron waves have affected countries around the world with different impacts [8].

The number of SARS-CoV-2 genome sequences in databases increased during 2022, associated with the unprecedented number of Omicron infections and with the consolidation of massive sequencing capabilities worldwide. However, even though more than 14 million SARS-CoV-2 genomes have been uploaded to databases until the present, only about 2.5% belong to South American countries [9], and most of them have been analyzed only for lineage assignment.

Moreover, there is a lack of studies on the important evolutionary aspects of different Omicron lineages and their circulation in different geographical regions, which could help to understand the potential impact of its emerging lineages in different epidemiological contexts.

This work aimed to study the dynamics of SARS-CoV-2 lineages during the third and the fourth waves of COVID-19 in Argentina, driven by Omicron, and to analyze their evolutionary pattern and behavior in light of the local epidemiological scenario.

## 2. Materials and Methods

### 2.1. Samples

**Figure 1:**
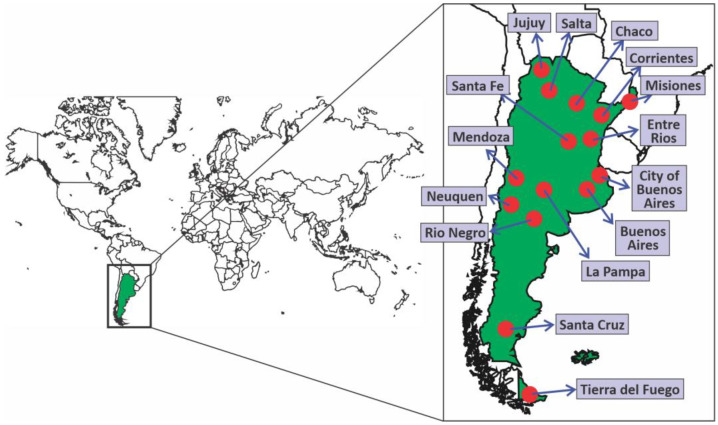
Sampling points for genomic surveillance of SARS-CoV-2 variants in Argentina.

Molecular surveillance was performed on a total of 3431 samples from the capital city (City of Buenos Aires) and 14 provinces of the country, including the most populated districts, distributed as follows: City of Buenos Aires (n = 874), and provinces of Buenos Aires (n = 655), Chaco (n = 104), Corrientes (n = 103), Entre Ríos (n = 10), Jujuy (n = 26), La Pampa (n = 11), Mendoza (n = 95), Misiones (n = 76), Neuquén (n = 395), Río Negro (n = 1), Salta (n = 51), Santa Cruz (n = 3), Santa Fe (n = 918) and Tierra del Fuego (n = 109) (Figure 1).

Samples were collected between epidemiological weeks (EW) EW44/2021 and EW31/2022, covering the third and fourth waves of the COVID-19 pandemic in Argentina.

Surveillance was carried out in a fraction of 2.5–60% of the total positive cases weekly detected in different healthcare centers of the country, depending on the epidemiological situation at each moment and the sequencing capacity of the Proyecto PAIS sequencing nodes at each location [10]. Samples corresponded to randomly selected cases with no epidemiological link among them or with international travel.

### 2.2. Sequencing

The surveillance strategy was based on: i. Sanger sequencing of a 965 bp region of Spike spanning amino acids 428 to 750 (2074 samples), corresponding to the PCR29 fragment of the CDC Sanger sequencing protocol that uses primers CDC-29 Fw: W1_29F_22847: 5′-TTACAGGCTGCGTTATAGCTTGG-3′ and CDC-29 Rv: W1_29R_23812_5′-TGCTGCATTCAGTTGAATCACC-3′ [11], which allows for the identification of signature mutations associated with VOCs and VOIs, as previously described [12]; ii. Complete genome sequencing, for which the ARTIC protocol with the “midnight” primer set was used [13,14] with Oxford Nanopore or Illumina platforms (1357 samples). Nucleotide sequences generated for this study can be found in the GISAID database (https://www.gisaid.org/) under the GISAID Identifier: EPI_SET_230114um.

### 2.3. Statistical Analysis

The frequencies of variant detection and their 95% confidence intervals (CI95%) were estimated with the Wilson/Brown method, implemented in the Graph Pad Prism v.8.3 program (San Diego, CA, USA, www.graphpad.com).

### 2.4. Phylogenetic Analysis

Phylogenetic analysis was carried out for Omicron lineages BA.1, BA.2 and BA.4/BA.5 and their descendants to confirm the lineage assignment and to study their introduction and spread in Argentina.

Datasets included the Argentine sequences of each lineage and reference sequences for different SARS-CoV-2 lineages. For BA.2 and BA.4/BA.5, analyses also included the five most closely related sequences (with less than 10 SNPs), selected using the AudacityInstant application in the GISAID EpiCoV© database (https://www.gisaid.org) on 14 August 2022 [9].

Alignments were built using MAFFT v7.486 [15] and maximum likelihood trees were built using IQ-TREE v.2.1 [16], using the nucleotide evolutionary model according to the Bayesian Information Criterion estimated using ModelFinder [17]. The Shimodaira–Hasegawa-like approximate likelihood ratio test (SH-aLRT, 1000 replicates) [18] was used to evaluate the reliability of the groups. For BA.2 and BA4/BA5 phylogenies, the Ultrafast bootstrap Approximation (UFB, 1000 replicates) [14] was also used.

We gratefully acknowledge the authors from the originating laboratories responsible for obtaining the specimens and the submitting laboratories where genetic sequence data were generated and shared via the GISAID Initiative, on which part of this research is based (GISAID Identifiers: EPI_SET_221215va, EPI_SET_221215gu, EPI_SET_221215wf).

### 2.5. Phylodynamic Analysis

The doubling time, the exponential growth rate and the time to the most recent common ancestor (MRCA) were estimated from the genome data for lineages BA.1, BA.2 and BA.4/BA.5 using an exponential growth coalescent model and the uncorrelated lognormal molecular clock in the BEAST v.1.10.4 software package [19].

For these analyses, datasets included Argentine sequences only from the exponential period for each lineage (i.e., the beginning of the sustained detection in surveillance analyses until the peak of registered cases in each wave or the peak of lineage frequency (Table S1)). To reach the convergence of the analyses in affordable times, given the high number of sequences of lineage BA.1, a subsampling of the exponential period of the third wave was done (covering from EW50/2021 to EW02/2022), with a final dataset for lineage BA.1 that included 138 sequences (GISAID Identifier: EPI_SET_221215ph). The dataset for lineage BA.2 included sequences from EW11/2022 to EW20/2022 (n = 173 sequences) (GISAID Identifier: EPI_SET_221215am), whereas the dataset for lineages BA.4/BA.5 (that were analyzed together) included sequences from EW20/2022 to EW30/2022 (n = 102 sequences) (GISAID Identifier: EPI_SET_221229hr).

The temporal structure of the datasets was assessed through the Root-to-tip analysis with TempEst v1.5.3 [20], for which a positive correlation between genetic divergence and sampling time is expected in datasets suitable for a phylodynamic analysis with tip dating calibration. However, this exploratory analysis failed to confirm the temporal structure for datasets of lineages BA.1 and BA.4/BA.5. Thus, a mean rate of evolution of 1.2 × 10^−3^ substitutions/site/year (s/s/y) was used to calibrate all analyses, with a lognormal distribution on the mean rate prior, truncated between 0.92 × 10^−3^ and 1.49 × 10^−3^, to cover the HPD95% intervals previously obtained for Omicron lineage BA.1 [21]. However, given that some temporal structure was observed for the dataset of lineage BA.2 (correlation coefficient ranged between 0.44–0.45 in the different functions of TempEst), another analysis using a tip-dating calibration was also carried out.

Analyses were run until convergence, assessed with an effective sample size higher than 200, and 10% of samples were discarded as burn-in. At least two runs were combined to summarize the posterior distribution of the parameters estimated.

### 2.6. Ethics Statement

The study was revised and approved by the Medical Ethics and Research Committees of ‘‘Ricardo Gutiérrez’’ Children’s Hospital, Buenos Aires, Argentina (DI-2020-165-GCABA-HGNRG). Informed consent was not obtained because patient information was anonymized and de-identified before analysis.

## 3. Results

### 3.1. Molecular Surveillance of SARS-CoV-2 Variants in Argentina

**Figure 2:**
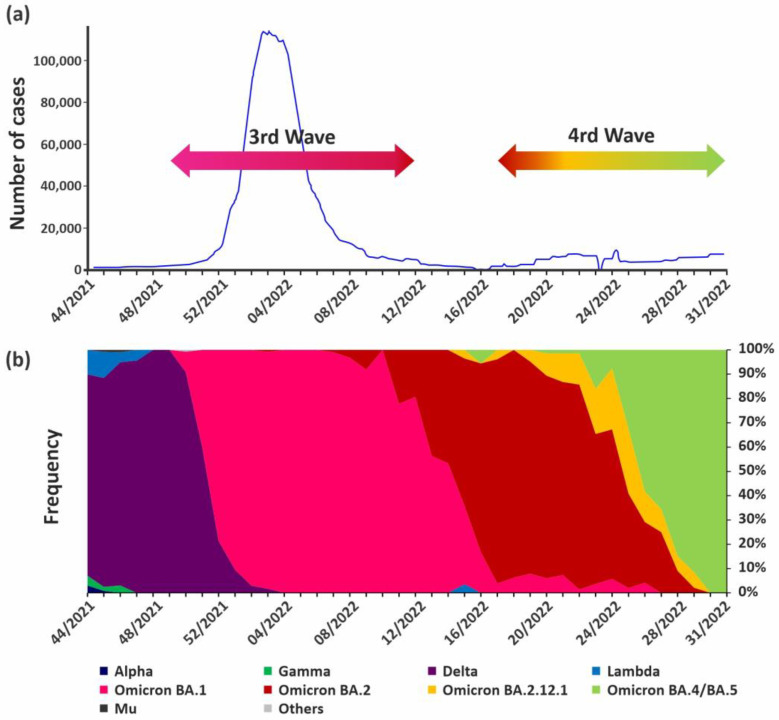
(**a**) Number of cases reported in Argentina from EW44/2021 to EW31/2022. Note that in EW15/2022 (between the third and the fourth waves), Argentina modified the COVID-19 massive testing criterion and since then, testing in public hospitals is only performed in populations under a higher risk for severe disease (>50 years or comorbidities). Data from https://ourworldindata.org/coronavirus#explore-the-global-situation, accessed on 1 December 2023. The arrows are colored according to the variant present at each moment of the wave. (**b**) Frequency of variant detection analyzed by epidemiological week, 2021–2022. Data from Spike and complete genome sequencing of samples from cases that did not present epidemiological link with travel (n = 3431).

The Omicron lineage BA.1 was first detected in local transmission cases in EW50/2021 (14 December 2021), reaching a frequency of 78.6% (CI95% = 72.6–83.6) two weeks later (EW52/2021), producing a third wave with a record number of cases in mid-January 2022 and fully displacing Delta by the end of January (EW04/2022) (Figure 2 and Table S1).

Except for two sporadic cases in EW03/2022, the Omicron lineage BA.2 was mainly detected since EW07/2022 (13 to 19 February) and became predominant in EW15/2022 (10 to 16 April) (60.7%, CI95% = 42.4–76.4), when cases of BA.2.12.1 were also detected. Lineage BA.2 and its sublineages displaced BA.1 more slowly than when BA.1 displaced Delta, which happened in only three weeks, from EW50/2021 to EW52/2022 (Figure 2 and Table S1).

The lineages BA.4/BA.5 have been detected since EW16/2022 (17 to 23 April), boosting the fourth wave together with BA.2 and its sublineages, becoming predominant by the end of June (EW26/2022) with a frequency of 58.3% (CI95% = 38.8–75.5) and reaching 100% of the new cases by the end of July (EW30/2022) (Figure 2 and Table S1).

### 3.2. Evolutionary Analyses of Omicron BA.1 and its Sublineages

**Figure 3:**
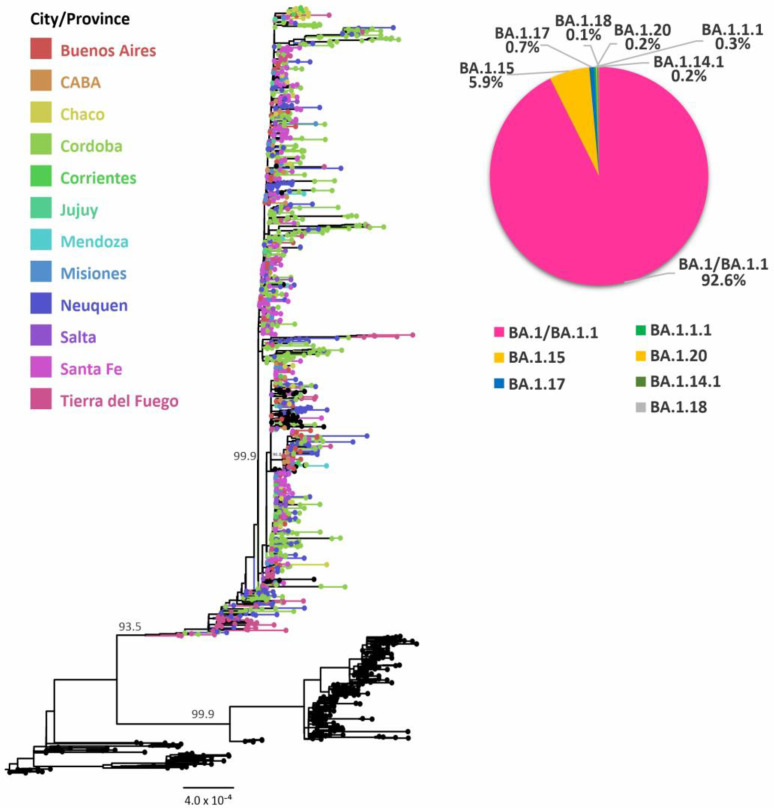
Phylogenetic tree of SARS-CoV-2 sequences focused on Omicron BA.1 and its sublineages. SH-aLRT support values are indicated at nodes for some groups. The branches and tips of the tree are colored according to the City or Provinces indicated in the legend. Black-colored tips represent sequences from other countries or sequences included as references for other lineages.

Lineage BA.1 and its sublineages were associated with the third wave of infections in Argentina, mainly driven by BA.1/BA.1.1 (91%); phylogenetic analysis did not separate BA.1.1 from BA.1, followed by BA.1.15 (5.9%) and other sublineages in minor proportions (Figure 3).

Phylodynamic analysis of the exponential phase of the BA.1 wave showed a doubling time of infections estimated in 3.3 days (HPD95% = 1.6–5.4), with an MRCA dated 12 November 2021 (HPD95% = 25 October–25 November) (Table 1).

### 3.3. Evolutionary Analyses of Omicron BA.2 and its Sublineages

**Figure 4:**
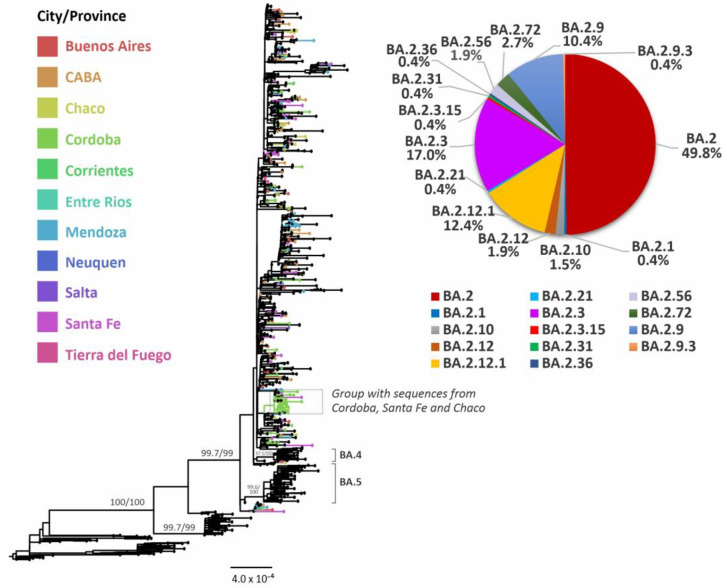
Phylogenetic tree of SARS-CoV-2 sequences, focused on Omicron BA.2 and its sublineages. SH-aLRT and UFB support values are indicated at nodes for some groups. The branches and tips of the tree are colored according to the City or Provinces indicated in the legend. Black-colored tips represent sequences from other countries or sequences included as references for other lineages.

Lineage BA.2 and its sublineages were associated with the early fourth wave of infections, mainly associated with lineages BA.2 (49.8%), BA.2.12.1 (12.4%), BA.2.3 (17.0%), BA.2.9 (10.4%), BA.2.72 (2.7%) and other sublineages in minor proportions (Figure 4). Notably, only one major monophyletic group from Argentinean sequences was observed including sequences from three provinces, whereas most of the other Argentine sequences were related to foreign sequences from several countries (Figure 4).

Phylodynamic analysis of the exponential phase of BA.2 circulation showed a doubling time of infections estimated in 9.9 days (HPD95% = 7.3–13.2) with an MRCA dated 3 February 2022 (HPD95% = 11 January–22 February) (Table 1).

In addition, when tip-dating calibration was used, the rate of evolution for the lineage BA.2 was estimated as 5.5 × 10^−4^ s/s/y (HPD95% = 3.1 × 10^−4^–7.7 × 10^−4^), with doubling times estimated in 16.5 days (HPD95%= 9.5–26.4), and the MRCA dated 24 November 2021 (HPD95% = 23 August–26 January) (Table 1).

### 3.4. Evolutionary Analyses of Omicron BA.4/BA.5 and its Sublineages

Lineages BA.4 and BA.5 were analyzed together for monitoring purposes and for phylodynamic estimations owing to their simultaneous introduction into the country, the inability of identifying them separately from the Spike sequencing strategy and their close phylogenetic relationship.

**Figure 5:**
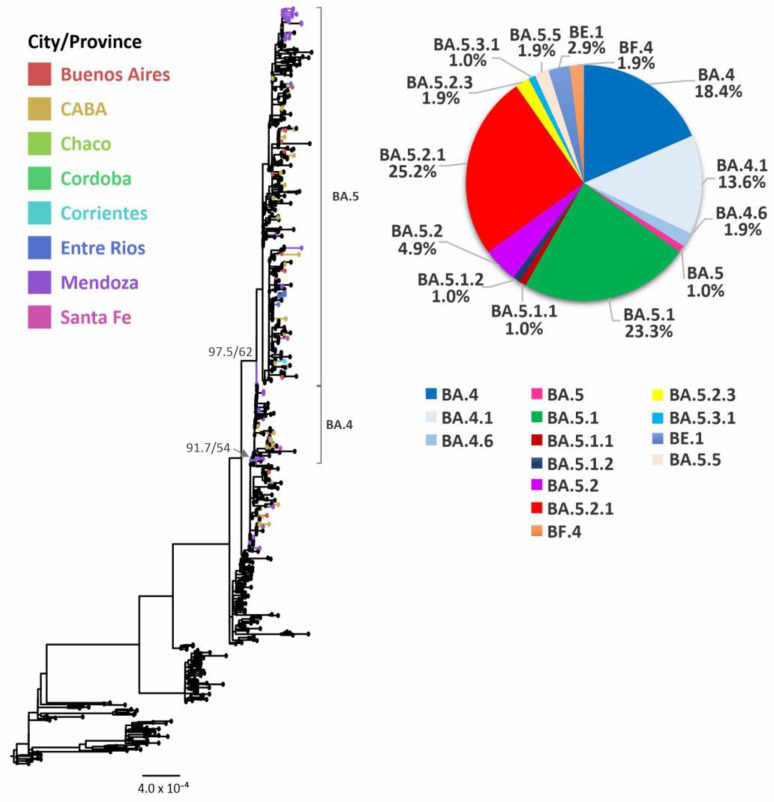
Phylogenetic tree of SARS-CoV-2 sequences, focused on Omicron BA.4/BA.5 and their sublineages. SH-aLRT and UFB support values are indicated at nodes for some groups. The branches and tips of the tree are colored according to the City or Provinces indicated in the legend. Black-colored tips represent sequences from other countries or sequences included as references for other lineages.

These lineages boosted the late fourth wave of infections into the country, mainly associated with lineages and sublineages BA.4 (18.4%) and BA.4.1 (13.6%), and with BA.5.1 (23.3%), BA.5.2 (4.9%), BA.5.2.1 (25.2%), BE.1 (2.9%) and other sublineages in minor proportions (Figure 5 and Table S1).

Similar to what was observed for lineage BA.2, Argentine sequences from several sublineages were intermingled with sequences from numerous countries and only a few local clusters were observed, showing a circumscribed geographical distribution, limited to one or two provinces (Figure 5).

The phylodynamic analysis of the exponential phase of infections caused by BA.4/BA.5 showed a doubling time estimated in 10.8 days (HPD95%= 6.1–17.3), with the MRCA dated 25 March 2022 (HPD95%= 15 February–24 April) (Table 1).

## 4. Discussion

The surveillance strategy implemented in Argentina allowed us to describe the introduction and establishment of the main SARS-CoV-2 Omicron lineages in the third and fourth waves of the COVID-19 pandemic in the country and analyze their different evolutionary dynamics.

Omicron waves worldwide were characterized by a very rapid expansion of several SARS-CoV-2 lineages that circulated almost without limits due to the absence of restrictions on international movement in the context of a massive application of vaccines, contrary to what happened during the dissemination of the previous circulating variants.

Before the emergence of Omicron, Delta was the dominant variant worldwide, whose introduction and establishment in Argentina were delayed in part due to an intense policy of international border controls implemented to reduce their impact while strengthening vaccination coverage in the population. For this and possibly other reasons, as a recent prior second wave, Delta was not associated with a COVID-19 wave in Argentina or other South American countries.

However, in December 2021–March 2022 (summer in the Southern hemisphere), Omicron BA.1 entered and caused the third wave of SARS-CoV-2 infections in Argentina. This wave was characterized by a very fast displacement of the Delta variant, showing a low level of clustering, with very similar viruses simultaneously infecting people worldwide. This was reflected in a relatively low number of lineages detected and in the intermingling of sequences from different Argentine provinces in the entire phylogeny. In other regions, the BA.1 epidemic wave has been traced back to a relatively small number of introductions with a later rapid expansion across the country, as was found in England [22].

By mid-April 2022, the COVID-19 testing criterion was modified, and tests were recommended and only enabled in public hospitals (at no cost to the patient) for prioritized groups, i.e., population with a higher risk for severe disease (>50 years or with comorbidities), which caused the registered number of cases to be incomparable to previous periods when testing was recommended and enabled in the public system for any symptomatic person. Considering that sequencing was mostly performed in samples from the public system, variant circulation proportions estimated before and after this change could have been affected by this distinct sampling, mainly oriented towards the prioritized population in the last period. As far as we know, there are no reports about a differential circulation of Omicron sublineages by age; however, due to the lack of age-stratified data, we were not able to perform a formal analysis on the influence of this factor.

Despite the change in the criterion and accessibility for COVID-19 testing, and consequently, in the case notification, the fourth wave was evidenced by an increase in the number of reported cases—although it was much lower than that observed in the third wave—and was initially associated with BA.2 and its sublineages.

Circulation of Omicron BA.2 in Argentina was characterized by the detection of several sublineages between EW03/2022 and EW29/2022 and very few clusters of Argentine sequences with a constrained distribution (one or few provinces), suggesting several introductions but limited transmission chains into the country.

Similarly, the sustained detection of Omicron BA.4/BA.5 was registered since EW20/2022, although, according to the results of phylogenetic analyses, these infections were mainly associated with several independent introductions and short transmission chains in the period analyzed in this work.

The evolutionary pattern of Omicron lineages detected in the third and fourth waves in Argentina reflects the distribution described in other countries from South America, the United States and Western Europe, as was observed in the pre-Omicron period [12]. However, this pattern differed from that of the first and second waves, when there was a high level of clustering and geographical structure within Argentina [12,23].

The introduction and establishment of SARS-CoV-2 lineages follow a complex pattern, balancing the intrinsic replication, immune evasion and transmission ability of viral variants, the waning immunity, and the policy of testing, reporting and case management of countries. Therefore, the epidemiological situation of countries could be determined by any of these factors, which could play a role in the variant dynamics.

In this work, the doubling time in the exponential period of the Omicron BA.1 wave in Argentina was estimated at 3.3 days, similar to what was observed in other regions [21,24], and was much faster than the estimations for BA.2 and BA.4/BA.5 in the country, which were estimated in about 10 days. In addition, ancestral times were also estimated from a subset of sequences that belongs to the period of the exponential growth of each lineage into the country. These ancestors were dated five (BA.1), six (BA.2) and eight (BA.4/BA.5) epidemiological weeks before their detection in local cases. These estimates along with the finding of multiple introductions suggest diversification outside Argentina and later entry into the country. For instance, for lineage BA.1, the ancestor for the Argentine sequences was dated to mid-November 2021, around one month after the time of the most recent common ancestor estimated from southern Africa sequences (mid-October 2021) [21].

The differences evidenced regarding the dynamics of Omicron waves could be due to several factors. The introduction of Omicron BA.1 into the country, displacing Delta, occurred in a context of unrestricted international travel and during the beginning of the presential meeting and social gatherings, which were discouraged a few months before. Omicron BA.2 (and later, BA.4/BA.5) also entered the country without restrictions, but occurred when it was still suffering the BA.1 wave (or the BA.2 wave); therefore, the populational immunity elicited post-wave may have limited the establishment of the new lineages.

On the other hand, the vaccination campaign against COVID-19 in Argentina started in January 2021 with the Sputnik V (Gam-COVID-Vac), ChAdOx1 nCoV-19 (Oxford-AstraZeneca), and BBIBP-CorV (Sinopharm) vaccines, initially for the prioritized population [25]. By the end of October 2021, 77.1% of the Argentine population received at least one vaccine dose and 58.4% completed the vaccination protocol [26]; however, for many people it had already been more than five months since the last dose, and the booster application of the vaccine only began in mid-November 2021. Moreover, in 2021, Argentina suffered the second wave caused by Gamma and Lambda variants [12], with the maximum number of infections in May 2021. Therefore, the Omicron lineage BA.1 entered a population with waned immunity (for prior infection or vaccination), whereas lineages BA.2 and BA.4/BA.5 enter a boosted population.

Different countries showed a distinct relative speed of replacement of BA.1 by BA.2, and of BA.2 by BA.4/BA.5 [27]. These differences must be analyzed considering each epidemiological situation and integrating several sources of information (for instance, vaccination, time to the previous waves, testing and contact tracing policies, molecular surveillance capacities, social acceptance of non-pharmaceutical interventions, etc.). Since the combination of these factors is almost unique for each country, the need for a local or regional analysis is mandatory for a better understanding of the driving factors.

Finally, some new sublineages derived from BA.2, BA.4 and BA.5 have recently emerged, adding immune evasion properties to those already reported in both vaccinated and priorly infected individuals [28,29]. Some of these emerging lineages have already become the new players in the scenario of SARS-CoV-2 circulation worldwide, whose impact will have to be analyzed in the near future.

## Figures and Tables

**Table 1:** Population dynamic parameters estimated from Bayesian coalescent analyses ^1^.

Lineage	Rate of Evolution [s/s/y] (HPD95%)	Ancestral Date (HPD95%)	Doubling Time [days] (HPD95%)	Exponential Growth Rate [days^−1^] (HPD95%)
BA.1	9.3 × 10^−4^ * (6.9 × 10^−4^–1.2 × 10^−3^)	12 November 2021 (25 October–25 November)	3.3 (1.6–5.4)	0.213 (0.127–0.441)
BA.2	1.0 × 10^−3^ * (8.7 × 10^−4^–1.1 × 10^−3^)	3 February 2022 (11 January–22 February)	9.9 (7.3–13.2)	0.070 (0.053–0.096)
	5.5 × 10^−4^ (3.1 × 10^−4^–7.7 × 10^−4^)	24 November 2021 (23 August –26 January)	16.5 (9.5–26.4)	0.042 (0.026–0.073)
BA.4/BA.5	8.5 × 10^−4^ * (6.4 × 10^−4^–1.0 × 10^−3^)	25 March 2022 (15 February–24 April )	10.8 (6.1–17.3)	0.064 (0.040–0.114)

^1^ Median values are informed. * These analyses were performed by calibrating with a mean rate of evolution and the sampling times for datasets considering only the exponential phase of infections of each lineage (see Section 2.5).

## Data Availability

Nucleotide sequences generated for this study can be found in the GISAID database (https://www.gisaid.org/), under the GISAID Identifier: EPI_SET_230114um.

## Supplementary Materials

The following supporting information can be downloaded at: https://www.mdpi.com/article/10.3390/v15020312/s1, Table S1: Frequency of SARS-CoV-2 variants in Argentina.
