# Supplementary material for: Omicron Waves in Argentina: Dynamics of SARS-CoV-2 Lineages BA.1, BA.2 and the Emerging BA.2.12.1 and BA.4/BA.5
Source: Viruses. 2023 Jan 22;15(2):312. doi: 10.3390/v15020312 (PMC9965068; doi:10.3390/v15020312)
Supplement: Supplementary file 1 [file viruses-15-00312-s001.zip › viruses-2163321-supplementary.pdf]

Table S1. Frequency of SARS-CoV-2 variants in Argentina.

EW[1]	Alpha			Gamma			Lambda			Delta			Omicron BA.1			Omicron BA.2			Omicron BA.2.12.1			Omicron BA.4/BA.5			Others			Total
	Freq (%)	CI95%[2]		Freq (%)	CI95%[2]		Freq (%)	CI95%[2]		Freq (%)	CI95%[2]		Freq (%)	CI95%[2]		Freq (%)	CI95%[2]		Freq (%)	CI95%[2]		Freq (%)	CI95%[2]		Freq (%)	CI95%[2]		
**44/2021**	**3.0**	0.8	8.5	**4.0**	1.6	9.9	**10.1**	5.6	17.6	**82.8**	74.2	89.0																**99**
**45/2021**	**0.8**	0.0	4.5	**1.7**	0.3	5.8	**10.7**	6.4	17.5	**86.0**	78.6	91.0													**0.8**	0.0	4.5	**121**
**46/2021**				**3.1**	0.8	8.6	**4.1**	1.6	10.0	**91.8**	84.7	95.8													**1.0**	0.0	5.6	**98**
**47/2021**							**4.4**	1.7	10.9	**95.6**	89.1	98.3																**90**
**48/2021**										**100.0**	96.7	100.																**114**
**49/2021**										**100.0**	97.1	100.																**129**
**50/2021**										**90.8**	84.9	94.5	**8.5**	4.9	14.3										**0.7**	0.0	3.9	**141**
**51/2021**										**59.9**	53.0	66.4	**40.1**	33.6	47.0													**202**
**52/2021**										**21.4**	16.4	27.4	**78.6**	72.6	83.6													**215**
**01/2022**										**9.6**	6.5	14.0	**90.4**	86.0	93.5													**240**
**02/2022**										**3.0**	1.4	6.3	**97.0**	93.7	98.6													**203**
**03/2022**										**1.7**	0.7	4.3	**97.4**	94.5	98.8	**0.9**	0.2	3.1										**232**
**04/2022**													**100.0**	98.2	100.0													**211**
**05/2022**													**100.0**	97.1	100.0													**129**
**06/2022**													**100.0**	96.1	100.0													**95**
**07/2022**													**99.0**	94.8	100.0	**1.0**	0.0	5.2										**105**
**08/2022**													**96.7**	88.8	99.4	**3.3**	0.6	11.2										**61**
**09/2022**													**91.8**	80.8	96.8	**8.2**	3.2	19.2										**49**
**10/2022**													**100.0**	91.4	100.0													**41**
**11/2022**													**77.8**	63.7	87.5	**22.2**	12.5	36.3										**45**
**12/2022**													**80.6**	63.7	90.8	**19.4**	9.2	36.3										**31**
**13/2022**													**56.3**	39.3	71.8	**43.8**	28.2	60.7										**32**
**14/2022**													**53.3**	36.1	69.8	**46.7**	30.2	63.9										**30**
**15/2022**							**3.6**	0.2	17.7				**32.1**	17.9	50.7	**60.7**	42.4	76.4	**3.6**	0.2	17.7							**28**
**16/2022**													**16.7**	5.8	39.2	**77.8**	54.8	91.0				**5.6**	0.3	25.8				**18**
**17/2022**													**3.8**	0.2	18.9	**92.3**	75.9	98.6	**3.8**	0.2	18.9							**26**
**18/2022**													**6.3**	1.1	20.1	**93.8**	79.9	98.9										**32**
**19/2022**													**7.9**	3.4	17.3	**87.3**	76.9	93.4	**4.8**	1.3	13.1							**63**
**20/2022**													**6.1**	2.4	14.6	**83.3**	72.6	90.4	**9.1**	4.2	18.4	**1.5**	0.1	8.1				**66**
**21/2022**													**7.4**	3.2	16.1	**79.4**	68.4	87.3	**11.8**	6.1	21.5	**1.5**	0.1	7.9				**68**
**22/2022**													**1.4**	0.1	7.7	**84.3**	74.0	91.0	**12.9**	6.9	22.7	**1.4**	0.1	7.7				**70**
**23/2022**													**3.7**	1.0	10.3	**61.7**	50.8	71.6	**18.5**	11.6	28.3	**16.0**	9.6	25.5				**81**
**24/2022**													**5.8**	1.6	15.6	**61.5**	48.0	73.5	**25.0**	15.2	38.2	**7.7**	3.0	18.2				**52**
**25/2022**													**2.0**	0.1	10.7	**38.8**	26.4	52.8	**26.5**	16.2	40.3	**32.7**	21.2	46.6				**49**
**26/2022**													**4.2**	0.2	20.2	**25.0**	12.0	44.9	**12.5**	4.3	31.0	**58.3**	38.8	75.5				**24**
**27/2022**																**25.0**	13.3	42.1	**9.4**	3.2	24.2	**65.6**	48.3	79.6				**32**
**28/2022**																**9.1**	3.1	23.6	**6.1**	1.1	19.6	**84.8**	69.1	93.3				**33**
**29/2022**																**2.1**	0.1	11.1	**6.4**	2.2	17.2	**91.5**	80.1	96.6				**47**
**30/2022**																						**100.0**	83.9	100.0				**20**
**31/2022**																						**100.0**	70.1	100.0				**9**
**Total**			**4**			**9**			**32**			**933**			**1718**			**480**			**80**			**172**			**3 **	**3431**

[1] Only cases from individuals without history of travel or close contact with travelers are included.

[2] The confidence interval of the frequency was estimated with the Wilson/Brown method, implemented in the Graph Pad Prism v.8.3 program (California, United States, www.graphpad.com).
